# Interleukin-5 Mediates Parasite-Induced Protection against Experimental Autoimmune Encephalomyelitis: Association with Induction of Antigen-Specific CD4^+^CD25^+^ T Regulatory Cells

**DOI:** 10.3389/fimmu.2017.01453

**Published:** 2017-11-01

**Authors:** Giang T. Tran, Paul L. Wilcox, Lindsay A. Dent, Catherine M. Robinson, Nicole Carter, Nirupama D. Verma, Bruce M. Hall, Suzanne J. Hodgkinson

**Affiliations:** ^1^Immune Tolerance Laboratory, UNSW Australia, Department of Neurology, Liverpool Hospital, Sydney, NSW, Australia

**Keywords:** parasite infection, Treg cells, interleukin-5, autoimmunity, experimental autoimmune encephalomyelitis

## Abstract

**Objective:**

To examine if the protective effect of parasite infection on experimental autoimmune encephalomyelitis (EAE) was due to interleukin (IL)-5, a cytokine produced by a type-2 response that induces eosinophilia. We hypothesize that, in parasite infections, IL-5 also promotes expansion of antigen-specific T regulatory cells that control autoimmunity.

**Methods:**

*Nippostrongylus brasiliensis* larvae were used to infect Lewis rats prior to induction of EAE by myelin basic protein. Animals were sham treated, or given blocking monoclonal antibodies to interleukin 4 or 5 or to deplete CD25^+^ T cells. Reactivity of CD4^+^CD25^+^ T regulatory cells from these animals was examined.

**Results:**

Parasite-infected hosts had reduced severity and length of EAE. The beneficial effect of parasitic infection was abolished with an anti-IL-5 or an anti-CD25 monoclonal antibody (mAb), but not anti-IL-4 mAb. Parasite-infected animals with EAE developed antigen-specific CD4^+^CD25^+^ T regulatory cells earlier than EAE controls and these expressed more *Il5ra* than controls. Treatment with IL-5 also reduced the severity of EAE and induced *Il5ra* expressing CD4^+^CD25^+^ T regulatory cells.

**Interpretation:**

The results of this study suggested that IL-5 produced by the type-2 inflammatory response to parasite infection promoted induction of autoantigen-specific CD25^+^*Il5ra*^+^ T regulatory cells that reduced the severity of autoimmunity. Such a mechanism may explain the protective effect of parasite infection in patients with multiple sclerosis where eosinophilia is induced by IL-5, produced by the immune response to parasites.

## Introduction

Correale and Farez observed that multiple sclerosis (MS) does not relapse in patients with eosinophilia from parasitic infection (1) and that eradication of parasites led to relapse of MS and loss of eosinophilia (2). The effect was demonstrated to be due to increased CD4^+^CD25^+^ T regulatory cells (Treg) (1) and regulatory B cells (3). Administration of parasites to modulate MS (4, 5) and other autoimmune diseases (6) has been trialed with limited effect (7). In these MS studies, the animal-derived parasites do not survive in humans so any effect of the parasites on the immune system was transient. There are major ethical issues with use of parasites that are human pathogens. This has led to attempts to identify antigens or chemicals in parasites that impede immunity and would not require infection with a pathogenic parasite, reviewed in Ref. (7, 8).

Hosts infected with parasitic worms can have reduced immune responses to autoantigens administered to induce autoimmunity, reviewed in Ref. (7) as well as vaccines (9) and infections, such as tuberculosis (10), malaria (11), HIV (12), and cholera (13). Thus, parasites of different species induce an immune inhibitory response to a variety of different antigens and pathogenic immune responses.

Parasitic infections induce a type-2 response that is initiated by innate lymphoid cells 2 (ILC2)-producing interleukin (IL)-5, IL-9, and IL-13 (14, 15), which induces CD4^+^ T helper 2 (Th2) cells that secrete IL-4, IL-5, and IL-13 (15). ILC2 and Th2 cells promote parasite expulsion (16). Parasites also induce Treg that can prevent immune-mediated elimination of parasites leading to chronic parasitic infections (9).

Diversion of T cell responses to a Th2 phenotype has been associated with a tolerogenic and less inflammatory immune response, including inhibition of pathogenic Th1 (17, 18) and Th17 (19) responses. Inflammatory responses to parasitic worms produce IL-5, which induces eosinophilia that in some models promotes parasite elimination (20) and in others prevent effective immunity (21, 22). The Th2 response to parasites also expands CD4^+^CD25^+^FOXP3^+^ Treg (23, 24) and other immune regulatory mechanisms, reviewed in Ref. (7, 8).

Experimental autoimmune encephalomyelitis (EAE), the animal model of MS, can be induced by immunization with autoantigen, which activates Th1 (25) and Th17 (26) effector responses, but a Th2 response is also induced and produces IL-4, IL-5, and IL-13 (25, 27, 28). Spontaneous recovery from EAE is associated with activation of Treg that promote recovery (29, 30). Parasitic infections reduce severity of EAE, including Th17 response and T cell infiltration to central nervous system (CNS) (31, 32). Parasitic infections that reduce severity of EAE and induce Treg include *Trypanosoma cruzi* (33), *Plasmodium chaubaudi* (34), *Trichinella spiralis* (35), and *Nippostrongylus brasiliensis* (36). Recently, it was reported IL-5 and IL-33 may mediate the protective effect of parasite infection on autoimmunity (36).

**Figure 1:**
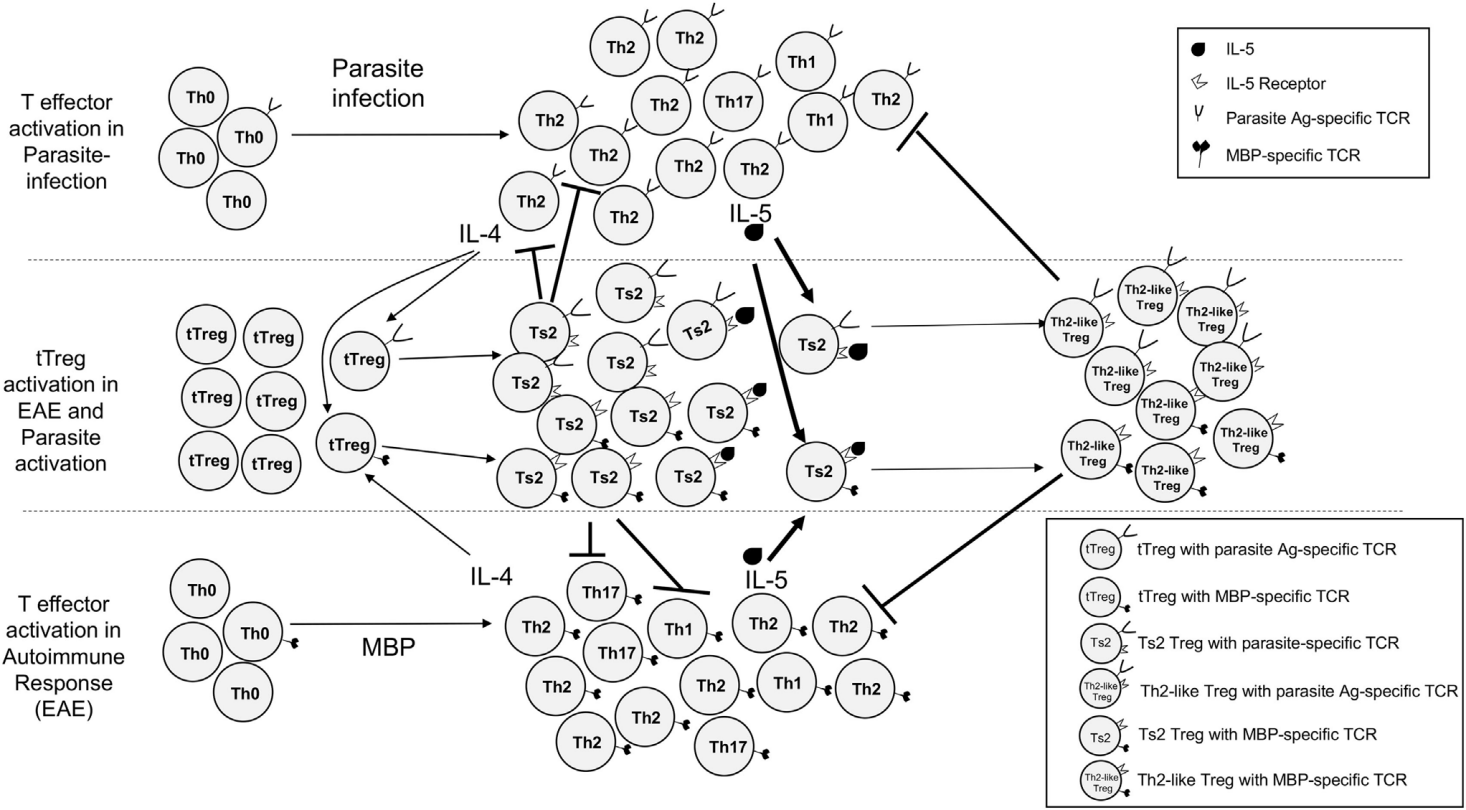
Proposed concurrent pathways of activation of effector CD4^+^CD25^−^FOXP3^−^T cells and CD4^+^CD25^+^FOXP3^+^ tTreg cells in parasite-infected hosts immunized with myelin basic protein (MBP) and complete Freund’s adjuvant to induce experimental autoimmune encephalomyelitis (EAE). Upper section shows response to parasites and lower section response to MBP. Effector CD4^+^ T cells with TCR that recognize the parasite antigens are predominantly activated to a type-2 response, with initial production of interleukin (IL)-4, then IL-5. Some Th1 and Th17 cells are activated by the parasites. IL-4 has two effects on tTreg. First, it polyclonally activates them like IL-2. Second, tTreg with TCR specific to the immunizing antigen are activated by IL-4 to proliferate and express IL-5Rα. CD4^+^ effector T cells that recognize autoantigen are activated to mainly Th1 and Th17 cells with a smaller T helper 2 (Th2) response. IL-2 also has two effects on tTreg. First, it both polyclonally expands them. Second, tTreg with receptor for autoantigen are activated to express receptors for Type-1 cytokines IFN-γ and IL-12 (pathway not illustrated here). These antigen-specific Th1-activated Treg usually control immune inflammation and lead to clinical recovery of EAE. The pathway we propose mediates the effect of parasite infection on EAE is induced by type-2 responses. IL-4 activates tTreg with TCR for autoantigen to expand and express IL-5Rα. These antigen-specific Treg suppress specific Th1 and Th17 responses, thus further polarizing the response to Th2 (19). Further stimulation with both specific antigen and IL-5, not IL-4, induces them to proliferate and differentiate into highly potent antigen-specific Th2-like Treg that inhibit the autoimmune response. Our hypothesis is that type-2 response to the parasite infection produces excess IL-4 and IL-5 that as a bystander effect promotes expansion of autoantigen-specific Treg that are dependent on IL-5 for their survival.

Our hypothesis is based on our observations about antigen-specific CD4^+^CD25^+^FOXP3^+^ Treg that mediate immune tolerance, reviewed in Ref. (37–39). These studies led us to recognize that antigen-specific Treg depend upon cytokines other than IL-2 and IL-4 (40–43) and that one such cytokine is IL-5 (43). This hypothesis is illustrated in Figure 1. The key finding related to this study is naïve CD4^+^CD25^+^FOXP3^+^ Treg activated with an autoantigen or alloantigen and rIL-4, not IL-2, are induced to express IL-5Rα, the specific receptor for IL-5 (19, 44).

Our hypothesis is supported by a number of findings in allo- and autoimmunity. First, we observed that treatment with IL-5 inhibits allograft rejection (45) and experimental autoimmune neuritis (19). Treatment with rIL-5 to suppress autoimmunity and allograft rejection depends upon host IL-4 (19, 45) and CD25^+^ T cells (19). The treatment with rIL-5 inhibits Th1 (19, 45) and Th17 responses (19). Second, we observed that animals tolerant to an allograft have CD4^+^CD25^+^FOXP3^+^ Treg that express IL-5Rα and respond to specific alloantigen in the presence of rIL-5 (46). Third, we observed that the CD4^+^ T cells from tolerant animals lose the capacity to transfer tolerance in culture unless they are activated by specific donor alloantigen and cytokines in supernatant from Con A-activated lymphocytes (40, 42). Cytokines that maintain these antigen-specific CD4^+^ tolerance transferring cells are IL-5 (43) and IFN-γ (42), whereas IL-2 (40) and IL-4 (41) do not.

Taken together, these findings show that naïve CD4^+^CD25^+^FOXP3^+^ Treg activated by a specific antigen can be activated down separate pathways by different cytokines. This is a two-step process. With type-1 responses, IL-2 and later IFN-γ or IL-12 activate these cells (44, 47). In type-2 responses, first IL-4 and later IL-5 activate antigen-activated Treg. The second step is blocked by the original activating cytokine, thus IL-2 (40) or IL-4 (41) do not maintain antigen-specific Treg and may inhibit them. These findings suggested IL-5 may promote these antigen-specific Treg. Thus, we reasoned that IL-5 produced by the inflammatory response to parasite may also expand autoantigen-specific Treg that have been first activated by IL-4 and the immunizing autoantigen.

In rats, *N. brasiliensis* infection induces eosinophilia and Th2 cytokines IL-4 and IL-5 between 7 and 14 days, after which these parameters return to normal by 28 days due to expulsion of the parasites (48). This study examined if the inflammatory response activated by infection with *N. brasiliensis* (49) produced IL-5 that tipped the balance to further expand antigen-specific activated Treg that had already been activated by autoantigen and IL-4, see Figure 1. Lewis rats were infected with *N. brasiliensis* (50) 3 days prior to immunization with myelin basic protein (MBP) and complete Freund’s adjuvant (CFA) to induce EAE. These parasite-infected hosts were treated with a monoclonal antibody (mAb) that inhibits IL-5 at the time of peak IL-5 production, which blocked the beneficial effects of parasite infections and reduced induction of antigen-specific Treg. To demonstrate the key role of IL-5, we examined whether therapy with recombinant (r) IL-5 inhibited EAE and induced antigen-specific Treg.

## Materials and Methods

### Induction of EAE

**Figure 2:**
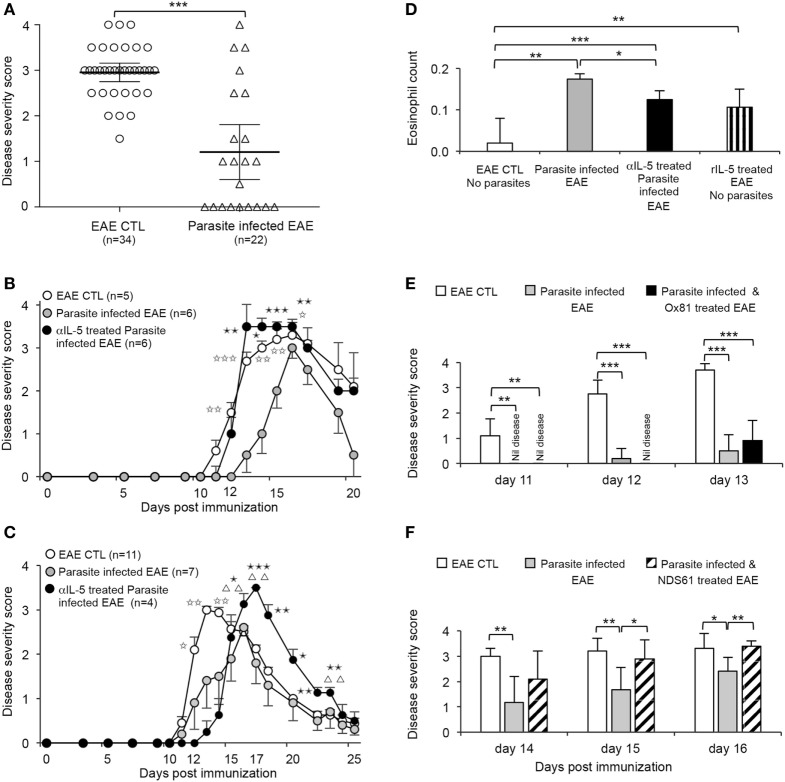
Effect of infection with *Nippostrongylus brasiliensis* on clinical course of experimental autoimmune encephalomyelitis (EAE) induced by inoculation of Lewis rats with myelin basic protein (MBP)/complete Freund’s adjuvant (CFA). **(A)** Clinical score of EAE at 13 days after immunization of all control Lewis rats immunized with MBP/CFA (○) (*n* = 34). All had developed clinical signs of EAE, with 33 having a score of >2. This was significantly more than in the parasite-infected EAE hosts (△) (*n* = 22) (*p* < 0.0001) in four of five experiments where parasitic infection had a significant effect on EAE. **(B)** Parasite-infected EAE rats (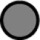) (*n* = 6) compared to untreated EAE controls (○) (*n* = 6), had slower onset of paralysis and the peak of disease was delayed; differences significant at day 12 (*p* = 0.01), day 13 (*p* = 0.001), day 14 (*p* < 0.005), day 15 (*p* < 0.01), and day 16 (*p* = 0.025). Treatment with a IL-5 blocking monoclonal antibody (mAb) starting at day 11 in parasite-infected EAE rats (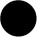) (*n* = 6) abolished the beneficial effects of parasite infection. The severity of EAE was similar to non-infected controls between days 11 and 16. Parasite-infected animals with EAE treated with anti-IL-5 mAb had more severe EAE than untreated parasite-infected EAE rats at day 13 (*p* = 0.01), day 14 (*p* < 0.05), day 15 (*p* = 0.001), and day 16 (*p* < 0.01). Results from one of three experiments. **(C)** In another experiment, anti-IL-5 mAb treatment of parasite-infected EAE rats (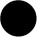) (*n* = 4) did not increase severity of EAE compared to parasite-infected EAE rats (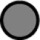) (*n* = 5) until day 16 (*p* < 0.05), day 17 (*p* = 0.001), day 18 (*p* < 0.01), day 20 (*p* < 0.05), day 21 (*p* = 0.01), and day 22 (*p* = 0.01). Parasite-infected rats had less severe EAE than non-infected EAE control rats (○) (*n* = 11) at day 12 (*p* < 0.05), day 13 (*p* < 0.005), and day 14 (*p* < 0.01). Anti-IL-5 mAb treatment significantly delayed recovery from EAE compared to controls with EAE, at day 16 (*p* < 0.01), day 17 (*p* < 0.005), day 22 (*p* = 0.01), and day 23 (*p* < 0.01). **(D)** Eosinophil counts at day 14 were increased in parasite-infected EAE hosts (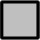) compared to EAE controls (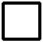) (** *p* < 0.01). Blocking IL-5 with anti-IL-5 mAb (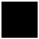) therapy reduced eosinophilia (*p* < 0.05) but it was still significantly greater than EAE controls (*p* < 0.0003). EAE rats treated with rIL-5 (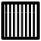)with 5,000 U daily i.p for 10 days starting on day of onset of clinical disease did not have significantly different eosinophil counts to parasite-infected EAE hosts but had significantly increased eosinophil numbers compared to EAE controls. **(E)** Anti-IL-4 mAb (MRCOx81) treatment of parasite-infected EAE rats (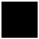) (*n* = 5) did not increase the severity of EAE, with similar disease scores to parasite-infected EAE rats with no treatment (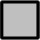) (*n* = 5) and significantly less than in control EAE (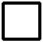) (*n* = 5). Data for days 11, 12, and 13 are shown (**p < 0.01, ***p < 0.001). **(F)** Depletion of CD25^+^ cells by treatment with an anti-CD25 mAb (NDS61) prior to immunization with MBP/CFA in parasite-infected EAE rats (▧) (*n* = 6) abolished protection conferred by parasite infection in EAE rats (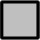) (*n* = 6), with no significant difference in disease severity scores to non-infected EAE controls (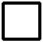) (*n* = 5). Anti-CD25 mAb-treated parasitic-infected EAE rats had more severe disease than parasite-infected EAE rats with no treatment, at day 15 (*p* = 0.003) and day 16 (*p* = 0.005).

Lewis and Dark Agouti (DA) rats were bred and maintained at the Animal House Liverpool Hospital. The foundations of these colonies were from the Animal Resources Centre, Canning Vale, WA, Australia. 10–12-week-old female rats were used in all experiments, and groups included littermates and animals from other litters. Experiments were approved by the Animal Ethics Committee of the UNSW Australia. Lewis rats were immunized with 10 mg of bovine MBP emulsified in CFA, as described (25, 28). DA rats were immunized with 20 mg DA rat spinal cord emulsified in CFA as described (51). Animals were monitored daily for weight loss and clinical disease was scored as 1+ limp tail, 2+ hind leg weakness, 3+ paraplegia, 4+ quadriplegia, 5+ death, as described (25, 28). In these studies, our immunization protocols induced clinical EAE in Lewis with disease onset by day 13 in all 34 rats in control groups (Figure 2A).

### Preparation of Autoantigens

Myelin basic protein was prepared from guinea pig spinal cord as described (25, 28). Spinal cord was prepared from exsanguinated DA rats, as described (51). Renal tubular antigen (RTA) was prepared from Lewis kidneys, as described (17). CFA used was incomplete Freund’s Adjuvant (Sigma-Aldrich, St. Louis, MO, USA) and 7 mg/ml of heat killed *Mycobacterium tuberculosis* H37 RA (Difco Laboratories, Detroit, MI, USA).

### Parasitic Infection with *Nippostrongylus brasiliensis* Larvae

*Nippostrongylus brasiliensis* was maintained in female Hooded Wistar rats at the University of Adelaide, SA, Australia with local ethics approval. Infective third stage larvae (L3) were prepared as described (52). Larvae were shipped to Sydney and washed three times in sterile PBS. Using an 18-G needle, 1,600 live L3 larvae in 100 µl of PBS were injected s.c. into the nape of the neck of each Lewis rat 3 days before immunization with MBP in CFA.

### mAb Therapy

TRFK5, anti–human IL-5 IgG1 mAb was obtained from Dr. W. Sewell, Center for Immunology, Darlinghurst, NSW, Australia. Although an anti-human IL-5 mAb, TRFK5 blocks rat IL-5 both *in vivo* and *in vitro* (19). MRCOx81, an anti–rat IL-4 mAb that blocks function of rat IL-4 (17) was a kind gift of Dr. Don Mason (MRC Cellular Immunology Unit, Dunn School of Pathology Oxford University, Oxford UK). TRFK5 and MRCOx81 were prepared as described (53) and given i.p. 7 mg/kg/dose every 4 days for 2 weeks. CD25^+^ cells were depleted with an anti-CD25 mAb, NDS61 (a kind gift of Dr. M. Dallman, Nuffield Department of Surgery, Oxford, United Kingdom) that was given i.p. at 7 mg/kg on days 4, 3, 2, and 0 before immunization, as described (54) (e-mail communication, Dr. G. Tellides, Department of Surgery, Yale University, New Haven, CT, USA) (19).

### Cytokines

Interleukin-5 was cloned and produced as relevant gene transfected Chinese hamster ovary-S (CHO-s) cell supernatant (55), characterized and quantified using bioassays in our laboratory, as described (17–19). Treatment with rIL-5 was 5,000 U daily i.p for 10 days, a dose that inhibits experimental autoimmune neuritis in Lewis rats (19).

### Lymphocyte Preparation and Separation

Cells were prepared from spleens and lymph nodes of Lewis or DA rats and immunofluorescence staining performed as described (19, 44). Anti-rat mAb used were R7.2 (TCR-αβ), G4.18 (CD3), MRCOx35 (CD4), MRCOx8 (CD8), MRCOx39 (CD25), MRCOx33 (CD45RA) (Pharmingen-BD, San Diego, CA, USA) and FITC anti-mouse/rat FOXP3 (eBioscience, San Diego, CA, USA). Lymphocyte subset analysis used a FACScan (BD, San Jose, CA, USA), or FACS Calibur as described (19, 44).

T cell subsets were enriched by indirect panning to deplete CD8^+^ T cells and B cells (CD45RA^+^) as described (19, 44, 56) to produce populations that were >98% CD4^+^, <1% CD8^+^ and <1% B cells. To enrich CD25^+^ cells, we used PE conjugated MRCOx39 and anti-PE magnetic beads using the MACS system (Miltenyi, Bergisch Gadenbach, Germany) as described (19, 44, 56). The CD4^+^CD25^+^ T cell populations were 90–96% CD25^+^.

### Culture of CD4^+^CD25^+^ T Cells with Autoantigen

Rat CD4^+^CD25^+^FOXP3^+^ T cells are slow to proliferate, and responses to a specific antigen are small, but can be increased by addition of cytokines, in this case rIL-5 (19, 44, 46, 56). Detecting proliferation that is specific requires special conditions that eliminate non-specific background proliferation. Cell culture medium used was RPMI 1640 (GIBCO, Grand Island, NY, USA) supplemented with 100 ng/ml penicillin, 100 U/ml streptomycin (Glaxo, Boronia, VIC, Australia), 2 mM l-glutamine, 5 × 10^−5^ M 2-mercaptoethanol (Sigma) and 20% Lewis or DA rat serum, as described (19). Rat sera produced low background stimulation and allowed cytokine mRNA analysis with no background in autologous controls, as described (56). Stimulator cells were prepared from irradiated syngeneic thymus cells (Lewis or DA) that had been pre-cultured as described (19, 56), with no antigen as a control, or specific autoantigen MBP or emulsified rat spinal cord or irrelevant autoantigen RTA. rIL-5 at 200 U/ml was added to examine if this cytokine enhanced the response to specific autoantigen; either bovine MBP for Lewis cells or DA spinal cord antigen for DA cells. Cultures were for 4 days at 37°C in 5% CO_2_.

Results of proliferation experiments were expressed as means and SDs of proliferation. For comparison of results from different experimental groups, data were normalized by calculation of the stimulation index, which was proliferation to a specific-antigen primed self-stimulator cell divided by proliferation to an unprimed self-stimulator cells, as described (56).

### RT-PCR

RNA extraction, cDNA synthesis, and semi-quantitative PCR were performed on a Rotorgene PCR machine (Corbett Research, Mortlake, NSW, Australia) as described (44, 57). Known primers for rat *Gapdh, Il2, Ifn*γ, *Il4, Il5* (58), *Ifngr, Il5ra, Foxp3, T-bet, Gata3* (44), and *Il17ra* (59), SYBR Green I and Hot Master Taq polymerase (Eppendorf AG, Hamburg, Germany) or SensiMix DNA kit (Bioline, Alexandria, NSW, Australia) were used as described. Gene copy number was derived from a standard curve run in parallel and normalized against *Gapdh* as described (44).

### Statistics

The clinical score on each day post-immunization were compared using a non-parametric Mann–Whitney *U* test and *p* < 0.05 was considered a significant difference. Area under the curve of clinical EAE were calculated according to the formula described by Fleming et al. (60) and expressed as mean ± SE. Other data were expressed as mean and SD and analyzed with an unpaired Student’s *t*-test.

## Results

### Effect of Infection with *N. brasiliensis* on Clinical Course of EAE and the Effects of Blocking IL-5

In our laboratory, induction of EAE by MBP/CFA is very reliable, with all 34 controls related to experiments described in this manuscript, developing clinical disease by day 13 (Figure 2A). This allowed confidence that the reduced and delayed course of EAE in parasite-infected hosts was an effect of the parasites.

*Nippostrongylus brasiliensis* infection 3 days before inoculation with MBP emulsified in CFA delayed and reduced the severity of EAE in Lewis rats in four of five experiments. The one of five experiments where parasites had no effect is not included. With the other four experiments, at day 13, there was a significant reduced severity of EAE (*p* < 0.0001) compared to controls (Figure 2A). Parasite infection induced eosinophilia (Figure 2D).

Given the variability of the rate and tempo of the effects of parasite infection on EAE, results of individual experiments are shown. In all four experiments where parasites reduced severity of EAE, treatment with an anti-IL-5 mAb commencing at 10 days after immunization significantly reduced the beneficial effect of parasitic infection.

In three experiments, anti-IL-5 mAb treatment reversed the delay in onset of EAE seen with parasite infection, and the severity of EAE was similar to or more severe than non-infected controls. One of these experiments (illustrated in Figure 2B) shows parasite infection delayed the day of onset of clinical disease, from 11 (11–12) days [median (range)] in EAE controls to 14 (11–16) days in parasite-infected animals (*p* = 0.034). In anti-IL-5 mAb-treated parasite-infected animals, the day of onset of clinical disease was similar to that of EAE controls, 12 (11–14) days. The area under the curve for clinical disease was less in parasite-infected animals (11.7 ± 1.6) compared to EAE controls (20.7 ± 2.4, *p* = 0.017) and anti-IL5 mAb-treated parasite-infected animals (18.0 ± 1.4; *p* = 0.026).

In one experiment, the effect of anti-IL-5 mAb treatment was delayed and the early course of EAE in these animals was similar to the untreated parasite-infected EAE group (Figure 2C). In this experiment, animals treated with anti-IL-5 mAb had a greater peak of disease and recovery was delayed compared to untreated EAE-parasite-infected rats and EAE controls (Figure 2C). The area under the curve of parasite-infected hosts (13.6 ± 2.8) was less than EAE controls (24.5 ± 3.1) (*p* < 0.05). Anti-IL-5 mAb-treated parasite-infected animals area under the curve was not different to EAE controls (17.0 ± 2.0). This delay in recovery seen in this experiment is consistent with anti-IL-5 mAb reducing the regulatory mechanisms that inhibit the immune response that allow recovery from EAE.

*Nippostrongylus brasiliensis* infection takes about 7 days to induce eosinophilia, so IL-5 appeared well after the activation of T cells to MBP. Furthermore, the parasites are expelled by 14 days after which IL-5 production rapidly drops.

Treatment with an IL-4 blocking mAb (MRCOx81), commencing at day 10 after immunization had no effect on the protective effect of parasitic infection (Figure 2E). This treatment served as a control for the anti-IL-5 mAb treatment. The lack of effect of anti-IL-4 mAb on the effect of parasites on EAE may be due to IL-4 produced by the early Type-2 response activating antigen-specific Treg to express IL-5Rα and become dependent on IL-5 not IL-4.

Treatment with NDS61 mAb to deplete CD25^+^ cells before immunization with MBP/CFA reduced the protective effect of parasitic infection on EAE (Figure 2F). This therapy reduced the number of CD4^+^FOXP3^+^ T cells in blood when compared to untreated controls (1.2–1.6% vs. 2.3–3.8%) at day 28. This showed CD25^+^ cells were required for the beneficial effects of parasitic infection.

### Comparison of CD4^+^CD25^+^ T Cells from Parasite-Infected and Control Rats with EAE

**Figure 3:**
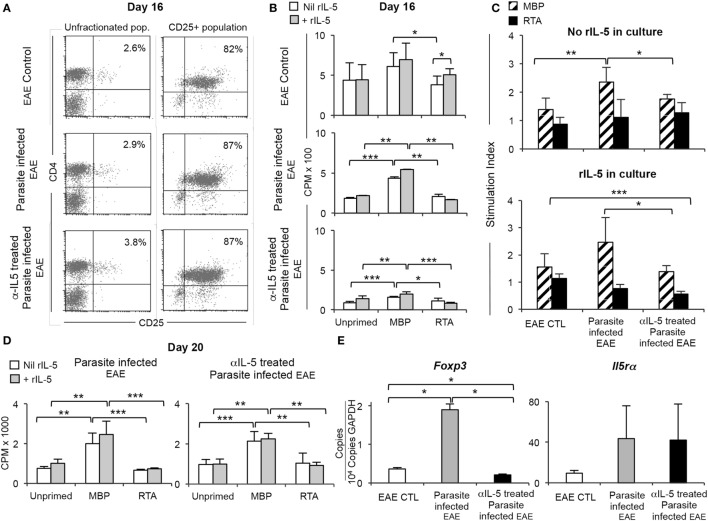
Comparison of CD4^+^CD25^+^ T cells from parasite-infected Lewis rats with experimental autoimmune encephalomyelitis (EAE), with and without anti-IL-5 monoclonal antibody (mAb) treatment. **(A)** FACS analysis of splenic T cells at 16 days post-immunization from EAE control rats and parasite-infected EAE rats treated with or without anti-IL-5 antibody (panels on left). The proportions of CD4^+^CD25^+^ T cells were not different to those from control EAE rats (2.6–3.8% vs. 2.5–3.9%). The CD4^+^CD25^+^ T cells were enriched in single step process to 82–87% of cells (right panel). These enriched samples were tested for proliferation in response to autoantigen **(B)** and expression of specific mRNA by RT-PCR **(E)**. **(B)** Proliferation of enriched CD4^+^CD25^+^ T cells [as described in **(A)**] in cultures with Lewis stimulator cells either unprimed (self) or primed with either myelin basic protein (MBP) or an irrelevant autoantigen renal tubular antigen (RTA). Cells were cultured in the presence (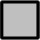) or absence (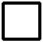) of rIL-5. Proliferation measured at 4 days by ^3^H-thymidine incorporation. *p < 0.05, **p < 0.01, *** *p* < 0.001. Cells from parasite-infected EAE rats had significantly higher proliferation to specific autoantigen, than to RTA or unprimed stimulators. There was much less proliferation of cells from anti-IL-5 mAb treated parasite-infected EAE rats. EAE controls had non-antigen-specific proliferation. **(C)** Data from 3B, expressed as stimulation indices. Response to specific-autoantigen MBP (▧) compared to that to third-party RTA (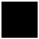). Upper panel shows stimulation indices of cultures without rIL-5 added CD4^+^CD25^+^ T cells from parasite-infected rats have higher stimulation indices than those from anti-IL-5 mAb treated parasite-infected hosts (*p* = 0.04) and EAE controls (*p* = 0.004). A stimulation index of 1 or less shows there is no antigen-specific effect. Thus, all populations did not respond to a third-party antigen, RTA. Lower panel shows stimulation indices in cultures with rIL-5 was added. The response to MBP-primed stimulator cells of CD4^+^CD25^+^ T cells from parasite-infected hosts was greater than that of cells from anti-IL-5 mAb-treated parasite-infected hosts (*p* = 0.03). There was no specific response to RTA by any cell population. Together, these results show that parasite-infected hosts develop more CD4^+^CD25^+^ T cells that respond to MBP, and this is abolished by anti-IL-5 mAb treatment. This suggests that IL-5 produced in response to the parasite infection enhances the generation of antigen-specific T regulatory cells. **(D)** Proliferation of enriched CD4^+^CD25^+^ T cells prepared as described in Figure 3A, from splenic cells taken at day 20 and cultured for 4 days with stimulator cells as described in **(B)** in the presence (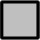) or absence (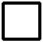) of rIL-5 (**p < 0.01, ***p < 0.001). Proliferation of cells from parasite-infected EAE rats and anti-IL-5 mAb-treated parasite-infected EAE was specific for MBP and similar, as all animals had recovered from EAE and had developed autoantigen-specific Treg. There was no response to third-party RTA-primed cells. **(E)** RT-PCR of mRNA from CD25^+^ T cells prepared as described in **(A)** from spleens collected at day 23. Expression of *Foxp3 and Il5ra* relative to *gapdh* in EAE controls, parasite-infected EAE, or anti-IL-5 mAb-treated parasite-infected EAE rats.

Spleen cells from parasite-infected and control EAE rats were analyzed at 16 days at the peak of clinical disease just before commencement of recovery. The percentage of CD4^+^CD25^+^ T cells in parasite-infected EAE rats had not increased above the normal range of 2–4% (Figure 3A, panels on left).

CD25^+^ T cells from control EAE rats and parasite-infected EAE rats treated with or without anti-IL-5 mAb were selected by a single step enrichment protocol without prior depletion of B cells and CD8^+^ cells. This cell population was 82–87% CD4^+^CD25^+^ (Figure 3A panels on right) with over 80% FOXP3^+^ (data not shown). Enriched CD25^+^ T cells were cultured for 4 days with irradiated Lewis stimulator cells that have been non-primed (self), MBP-primed, or primed with an irrelevant autoantigen RTA (Figures 3B,C). Cultures supplemented with rIL-5 were compared to those with no cytokine added. CD25^+^ T cells from parasite-infected EAE rats responded to MBP-primed but not to non-primed (*p* < 0.001) or RTA-primed (*p* < 0.01) stimulator cells. With addition of IL-5 to these cultures, there was a small enhancement of the response to MBP-primed stimulator (*p* < 0.05) but not to unprimed or RTA-primed stimulator cells. CD25^+^ T cells from EAE controls rats responded in a non-antigen-specific manner, although the response to MBP was greater than RTA (*p* < 0.05). IL-5 did not enhance the response to MBP (Figure 3B). The reason for this higher non-specific background response of CD4^+^CD25^+^ T cells from EAE controls was not examined, but may relate to the EAE control rats being at the peak of clinical disease, with no inhibitory effect from parasites.

As each test cell population has different levels of non-specific proliferation, the stimulation index was used for comparison between the three groups (Figure 3C). A stimulation index of 1 or less is no specific proliferation to that antigen. There was a marked autoantigen-specific (MBP) response of CD4^+^CD25^+^ T cells from parasite-infected animals that was significantly greater than that of CD4^+^CD25^+^ T cells from either parasite-infected EAE rats treated with anti-IL-5 mAb (*p* = 0.04) or EAE controls (*p* = 0.006). CD4^+^CD25^+^ T cells from both EAE controls and from anti-IL-5 mAb-treated animals had a modest response to MBP-primed stimulator cells. CD4^+^CD25^+^ T cells from all groups had no specific response to third-party RTA-primed stimulator cells with stimulation indices of around one.

In cultures with rIL-5 added, the response of CD4^+^CD25^+^ T cells from parasite-infected hosts was greater than that of cells from parasite and anti-IL-5 mAb-treated hosts (*p* = 0.03) but not to that of cells from EAE controls. rIL-5 had no effect on the lack of response to third-party RTA.

These results showed that at day 16, the CD4^+^CD25^+^ T cells from parasite-infected hosts had a significant specific response to MBP, which was not much greater than that with cells from EAE controls or in parasite-infected controls treated with anti-IL-5 mAb. This was consistent with the parasite infection *via* induction of IL-5 production, accelerating induction of antigen-specific CD4^+^CD25^+^ T cells.

At day 20, when animals had recovered from EAE, both parasite-infected groups whether treated with anti-IL-5 mAb or not, had a specific response to MBP that was significantly greater than to RTA and unprimed stimulator cells (Figure 3D). Taken together, these results suggest parasitic infection brings forward the development of antigen-specific Treg.

CD25^+^ Treg from parasite-infected animals expressed more *Il5ra* than those from control EAE rats (Figure 3E). *Foxp3* expression by CD4^+^CD25^+^ T cells was greatest in the parasite-infected group, and less in the anti-IL-5 mAb-treated parasite-infected and control EAE groups. This was consistent with the expansion of the IL-4-induced antigen-specific CD4^+^CD25^+^ Treg that express *Il5ra* (19, 44).

### Effect of Treatment with rIL-5 on Clinical Course of EAE

**Figure 4:**
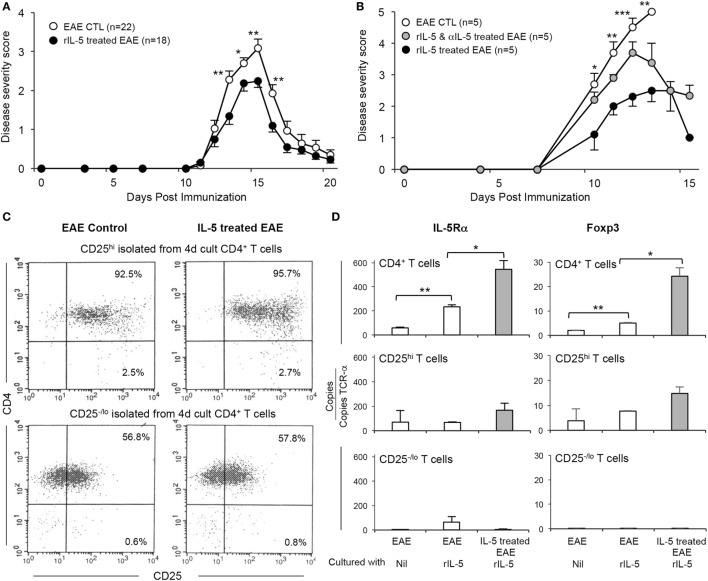
Effect of therapy with rIL-5 on clinical experimental autoimmune encephalomyelitis (EAE) and induction of interleukin (IL)-5Ra expressing Treg. **(A)** Clinical course of EAE in Lewis rats treated with 5,000 U of rIL-5, daily from onset of clinical disease (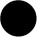) (*n* = 25), compared to EAE in sham-treated controls (○) (*n* = 18). From 2 days after rIL-5 treatment commenced, rats had less severe disease than in controls: day 13 (*p* < 0.005), day 14 (*p* = 0.05), day 15 (*p* < 0.005), and day 16 (*p* < 0.005). Both groups had recovered by 17 days. **(B)** Clinical course of EAE in Dark Agouti DA rats immunized with autologous spinal cord in CFA and treated with 5,000 U of rIL-5, daily from day 8 at onset of clinical disease (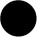) (*n* = 5) compared to EAE in sham-treated controls (○) (*n* = 5) and those treated with a combination of rIL-5 and anti-IL-5 mAb from day 8 (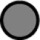) (*n* = 5). rIL-5-treated animals compared to sham-treated controls had a significantly milder disease at day 10 (*p* < 0.05), day 11 (*p* < 0.05), day 12 (*p* = 0.001), and day 13 (*p* < 0.01). All EAE controls had severe disease and either died or were euthanized at day 13. Anti-IL-5 mAb abolished the benefits of rIL-5 treatment at 10–12 days. **(C)** FACS analysis of enriched CD4^+^CD25^hi^ and CD4^+^CD25^−/lo^ splenic T cells from DA rats with EAE, with or without IL-5 treatment. Spleen and lymph nodes cells were recovered at 14 days and then cultured for 4 days with spinal cord antigen in the presence or absence of rIL-5 before they were enriched for CD4^+^CD25^hi^ and CD4^+^CD25^−/lo^ populations. **(D)** RT-PCR for il5ra and foxp3 expression on CD4^+^ T cells isolated from DA rats and cultured as described in Figure 4C. Results expressed as copies of relevant gene per TCR-α copy number for that specimen to normalize for T cell number. CD4^+^ and CD4^+^CD25^+^ T cells from rIL-5 treated hosts had greater *il5ra* and *foxp3* expression than those from control EAE not treated with IL-5. Populations depleted of CD4^+^CD25^hi^ T cells had minimal *il5ra* and *foxp3*, even if cultured with rIL-5.

Given that blocking IL-5 eliminated the effects of parasitic infection on EAE, we examined if treatment with rIL-5 alone modified the course of EAE (Figure 4A). Lewis rats with EAE were either treated with rIL-5 or supernatant from non-transfected CHO-s cell culture commencing at day 11, just before onset of clinical EAE. A dose of 5,000 U of rIL-5 per day was given for 10 days as this dose markedly reduced nerve demyelination in experimental autoimmune neuritis (19). rIL-5 therapy reduced the severity of EAE within 2 days. EAE was less severe with rIL-5 treatment at day 13 (*p* < 0.005), day 14 (*p* < 0.05), day 15 (*p* < 0.005), and day 16 (*p* < 0.005). By day 17, both groups had recovered (Figure 4A). These are combined results from four separate experiments. The area under the curve was less in rIL-5 treated (8.9 ± 0.9) (*n* = 22) than in control EAE (15.7 ± 1.9) (*n* = 18) (*p* = 0.002).

To examine if the IL-5 effect was present in strains other than Lewis rats, a second model of EAE was used. DA rats were immunized with autologous spinal cord in CFA was used. rIL-5 treatment was given from day 8 post-immunization, a few days before the onset of clinical disease and continued daily for 10 days (Figure 4B). The severity of EAE was reduced in rIL-5 treated rats compared to sham-treated controls at days 10 and 11 (*p* < 0.5), day 12 (*p* < 0.005) and day 13 (*p* < 0.01). Sham-treated EAE controls had severe disease and were euthanized at day 13, as required by our animal ethics approval (Figure 4B). Animals treated with both rIL-5 and anti-IL-5 mAb from day 8 post-immunization had lower clinical paralysis scores than controls at days 10, 11, and 12 and later recovered. The area under the curve was less for IL-5 treated (10.8 ± 2.1) than for EAE untreated controls (22.2 ± 0.6) (*p* = 0.008). This was one of four experiments that showed a similar effect of rIL-5 therapy on EAE in DA rats.

CD4^+^ T cells from the spleens of rIL-5-treated and sham-treated DA rats were compared by RT-PCR either as freshly isolated or after 4-day culture with DA rat spinal cord autoantigen. Cultures were either supplemented with IL-5 or had no supplement with a cytokine. The cultured CD4^+^ T cell population was separated into CD4^+^CD25^hi^ T cells (>95% enriched) and CD4^+^CD25^lo^ T cells and subjected to FACS (Figure 4C) and RT-PCR to determine gene expression profiles.

Freshly isolated CD4^+^ T cells from IL-5 treated EAE animals had more *Foxp3, Il5ra, Il4, Il5*, and *Gata3* than sham-treated EAE controls (data not shown). There was no significant difference in expression of Th1-associated genes *Il2, Ifng*, and *Tbet*, nor the Th17 gene *Il17* (data not shown).

After culture with rIL-5, both the unseparated CD4^+^ T cells and CD4^+^CD25^hi^ T cells from rIL-5-treated EAE rats expressed more *Foxp3* and *Il5ra* mRNA than cells from sham-treated EAE controls (Figures 4C,D). By contrast, the CD4^+^CD25^−^/^lo^ T cells had very low expression of *Foxp3* and *Il5ra* (Figure 4D).

Thus, rIL-5 therapy alone reduced severity of clinical EAE, accelerated recovery and increased the proportions of *Il5ra* and *Foxp3* expressing CD4^+^CD25^+^ Treg. This confirmed that the effects of parasite infection could have been mediated by IL-5 alone.

## Discussion

The beneficial effects of environmental acquired parasitic infections on MS and other autoimmune diseases have been associated with eosinophilia. Induction of eosinophilia is due to Type-2 cytokines IL-4 and IL-5 produced by Th2 and ILC2 cells (14, 15) in response to the parasitic infection. These clinical observations led to studies where patients with MS or inflammatory bowel disease are infected with parasites (6, 61–65). Deliberate infection of patients with a parasite has significant ethical issues, as parasites that infect humans can induce morbidity and mortality. In clinical studies to date, non-human parasites were given to patients, but these are rapidly eliminated and do not produce a prolonged Type-2 response and eosinophilia (6, 61–65). This may explain why they failed to show a therapeutic benefit. As a consequence, there are many investigations of molecules produced by parasites that alone may induce the Type-2 response and eosinophilia, reviewed in Ref. (7, 8). Administration of enriched parasitic molecules that induce Type-2 responses and eosinophilia may be therapeutic without the risks associated from persistent parasitic infection.

As tissue invasive parasitic infection induces type-2 responses that produce IL-4, IL-5, and IL-13 (66, 67), and IL-5 induces eosinophilia (21), these observations led us to the hypothesis examined in this study. We previously observed that IL-5 promotes induction of antigen-specific CD4^+^CD25^+^ Treg that prevent autoimmune-mediated demyelination in experimental autoimmune neuritis (19). We also demonstrated that naïve CD4^+^CD25^+^FOXP3^+^ Treg activated by a specific antigen and the Type-2 cytokine IL-4, but not the usual Treg cytokine IL-2, are induced to express IL-5Rα (44) and that IL-5 further activates these antigen-specific Treg (44). Antigen-specific Treg that express *Il5ra* are further expanded by IL-5 and stimulation with specific antigen both *in vitro* (46, 56) and *in vivo* (19). We proposed that tTreg activated by a specific antigen and IL-4 require IL-5 to promote their survival (43) and their inhibition of auto- and allo-immunity (19, 43, 44), as illustrated in Figure 1.

In this study, we examined if the effects of parasitic infection could be due to increased IL-5, which not only induce eosinophilia but also as a bystander effect further expands antigen-specific CD4^+^CD25^+^ Treg activated by autoantigen and IL-4. The observations that supported our hypothesis were as follows: (i) infection with the parasitic nematode *N. brasiliensis* reduced the severity of EAE and induced eosinophilia, (ii) treatment with an anti-IL-5 mAb blocked this protective effect of parasitic infection and partially reduced the level of eosinophilia; (iii) compared to EAE controls and anti-IL5 mAb-treated parasite-infected EAE hosts, parasite-infected hosts with EAE had increased numbers of CD4^+^CD25^+^ T cells that responded to the specific-autoantigen MBP, but not to a third-party autoantigen; (iv) depletion of CD25^+^ cells prior to induction of EAE prevented parasitic infection reducing the severity of EAE. Treatment with an anti-CD25 mAb that depletes CD25^+^ cells is a standard method of showing CD4^+^CD25^+^ Treg are required to mediate the effect.

To test Koch’s postulate, we showed treatment with IL-5 reduced the severity of EAE to a degree comparable to parasite infection. We also showed IL-5 treatment, as with parasitic infection, increased the number of CD4^+^CD25^+^ Treg that expressed *Il5ra*.

These findings show parasitic infection mediates part of its effect of reducing the severity of EAE *via* Th2 and/or ILC2 cells (14, 15) activated to produce high levels of IL-5. IL-4 produced early in the response to parasitic infection may have contributed to the initial activation of antigen-activated Treg to induce expression of *il5ra* and subsequent dependence on IL-5.

When first described, Th2 cells were considered anti-inflammatory and tolerance-promoting (67). Nasal therapy with MBP peptides combined with IL-4 reduces severity of EAE in Lewis rats (68). Combining rIL-4 and anti-CD3 mAb therapy reduces severity of EAE and inhibits Type-1 responses, but spares type-2 (25). rIL-4 therapy prevents autoimmune nephritis (17). IL-10 therapy inhibits EAE, but its effect is abrogated by IL-4 (69). In IL-5 knock out mice, the severity and time of onset of EAE was similar to wild-type controls, but type-2 responses were reduced (70). The effects of parasite infection on EAE in IL-5 knock out animals are not known. On the other hand, ERK1 knockout animals have enhanced Th1 responses and reduced IL-5 production, and develop more severe EAE (71) consistent with type-2 responses modifying EAE. These studies do not prove type-2 cytokines directly inhibit autoimmunity, however.

Recently, Finlay et al. described that immunization with *Fasciola hepatica* secretory/excretory products inhibited EAE and reduced Th1 and Th17 responses (36). This effect is associated with type-2 responses, but is not dependent on IL-4, IL-10, or Treg. They identified that IL-33 with IL-5, produced by ILC2, mediates the effect by induction of eosinophilia (36). In this study, blocking IL-5 also reduced parasite-induced protection against EAE (36). IL-33 has been shown to reduce EAE by inhibition of Th1/Th17 response, as well as enhanced Type-2 responses and M2 macrophages (72). IL-33 enhances both Th2 effector responses and activation of Treg by Type-2 cytokines (73, 74), thus these findings may complement those described here. Other immune regulatory mechanisms may be induced by type-2 responses, including alternate activated macrophages (M2), which are induced when parasite infection reduces severity of EAE (32).

Although the induction of EAE is by Th1 and Th17 responses, there is induction of a Th2 response (25, 27). The recovery from EAE is driven by induction of antigen-specific CD4^+^CD25^+^ Treg and is dependent upon IFN-γ (75). This is the Th1 cytokine-induced pathway for activation of antigen-specific Treg (44, 47) that is the major contributor to the spontaneous recovery from EAE in controls (75).

The detection of the slow and small proliferation of antigen-specific Treg requires culture techniques that eliminate non-specific background proliferation, as we have previously described (19, 44, 46, 56). In this study, CD4^+^CD25^+^FOXP3^+^ Treg proliferation to the specific antigen MBP was greater before recovery from EAE (day 16) in parasite-infected hosts, than EAE controls and anti-IL-5 mAb-treated parasite-infected EAE hosts. This response was only to specific-antigen MBP and not to RTA or unprimed self-stimulator cells, confirming this was an antigen-specific Treg response. This confirmed that parasite-infected hosts had more MBP-specific CD4^+^CD25^+^ Treg than EAE controls and anti-IL-5 mAb-treated EAE animals. Further CD4^+^CD25^+^ T cells from parasite-infected and IL-5 treated hosts expressed more *Il5ra* than CD4^+^CD25^+^ T cells from EAE controls or anti-IL-5 mAb-treated parasite-infected EAE animals.

Our interpretation of the effects of parasitic infection is that production of IL-4 and IL-5 by the activated Th2 cells and ILC2 accelerates the induction of autoantigen-specific Treg that express *Il5ra*, which in turn, reduced the severity of EAE. The source of the IL-5 in our hosts was not identified but could be Th2 or ILC2 cells that have been activated in response to parasitic infection (36). Late treatment with an anti-IL-4 mAb did not prevent the effects of parasites on EAE. We think that by the time anti-IL-4 mAb was given, the host ILC2 and Th2 cells had already produced sufficient IL-4 to activate naïve CD4^+^CD25^+^FOXP3^+^ Treg with TCR specific for MBP to express *Il5ra*. In an allograft tolerance model, we showed IL-4 does not sustain the alloantigen-specific Treg that mediate tolerance (41), but IL-5 does (43). In other models, early blocking of IL-4 prevents induction of *il5ra* expressing Treg and rIL-5 therapy is ineffective (19, 45). With parasitic infection and rIL-5 therapy, these autoantigen and IL-4 activated Treg were further expanded, respectively, by parasite infection-induced host type-2 response producing IL-5 or by administered IL-5.

Whether or not the benefits of chronic parasitic infection where numbers of CD4^+^CD25^+^ T cells are increased in patients with MS (1, 2) is mediated in part or mainly by IL-5 requires investigation.

Historically, IL-5 has been considered pro-inflammatory in allergic diseases including asthma. IL-5Rα is not expressed by T cells other than IL-4 and antigen-activated CD4^+^CD25^+^FOXP3^+^ Treg (19, 44). IL-5Rα is mainly expressed on eosinophils, mast cells, and basophils, as well as a subpopulation of naïve B cells. Thus, IL-5 cannot activate T effector cells. Trials with an anti-IL-5 mAb in asthma showed blocking IL-5 had limited or no effect, except in patients with high eosinophil counts, reviewed in Ref. (76). Many patients, including those with parasitic infection have continued production of IL-5, which does not enhance autoimmunity or allergic diseases. The safety issues for IL-5 or IL-5 analogs in therapy will require careful examination, but may be acceptable. For treatment of chronic diseases such as MS, continued treatment may be required as the antigen-specific Treg require exposure to IL-5 to maintain their function.

The hygiene hypothesis related to autoimmunity and allergy can in part be explained by lack of parasitic infections that can reduce unwanted immunity, including allergies and autoimmunity (77). This study suggests IL-5 produced in response to parasite infection may mediate a major part of the protection from autoimmunity and allergy by activation of antigen-specific Treg that have been activated by IL-4.

High levels of IL-5 and the resultant eosinophilia are protective against some parasite species, but not against others and may even be counterproductive (21, 22). The immune response that eliminates parasites is complex and varies depending on the species of parasites, as well as the type-2 response and eosinophilia (78).

Our hypothesis is illustrated in Figure 1. Parasitic infection initially activates ILC2 cells producing IL-4, IL-5, and IL-13 (14, 15), which direct effector cells to differentiate into Th2 cells. Th2 cells initially produce IL-4 and later produce IL-5 and IL-13. In parallel, naïve tTreg activated by an antigen in the presence of high IL-4 are activated to express *Il5ra*. Treg activated by the parasite will suppress effector responses directed at the parasite. In our experiments, there is a second immune response to MBP, which activates Th17, Th1, and Th2 responses. Normally, the Th17 and Th1 response dominate, and IL-2 induces naïve CD4^+^CD25^+^ Treg activated by MBP to express *Ifngr* and *Il12rb2*. This produces potent antigen-specific Treg that control the effector response to MBP so clinical disease abates and animals recover from paralysis. This response is normally driven by IFN-γ (75). However, immunization with MBP/CFA also activates some effector Th2 cells that initially produce IL-4 (25, 27) and this promotes activation of antigen-activated Treg to express *Il5ra*. In the parasite-infected host, the IL-4 from the early type-2 response to parasites would increase the number of MBP-specific Treg that express *Il5ra* and these would be further expanded by IL-5 produced by the late type-2 response to the parasite. These MBP-activated Treg suppressed the response to MBP and reduced the severity of EAE.

The results as a whole are consistent with IL-5 that is produced in excess by the type-2 response to the parasites, promoting earlier expansion of autoantigen-specific Treg that limit the severity of immune injury in EAE. The identification of IL-5 as a key molecule in reducing immunity to autoantigens, some infections, vaccines, and exacerbating responses to allergens, makes IL-5 a target to block and restore protective or less destructive immunity.

## Author Contributions

GT designed and conducted experiments and contributed to analysis, PW conducted and analyzed experiments, LD produced and inoculated parasites and contributed to analysis, NV conducted experiments and contributed to analysis. CR conducted and analyzed experiments, NC conducted some control EAE experiments, BH designed and analyzed experiments, SH designed and analyzed experiments. All authors contributed to the writing of the manuscript.

## Conflict of Interest Statement

The authors declare that the research was conducted in the absence of any commercial or financial relationships that could be construed as a potential conflict of interest.
